# Novel *BRAF* alteration in desmoplastic infantile ganglioglioma with response to targeted therapy

**DOI:** 10.1186/s40478-018-0622-1

**Published:** 2018-11-05

**Authors:** Melissa M. Blessing, Patrick R. Blackburn, Jessica R. Balcom, Chandra Krishnan, Virginia L. Harrod, Michael T. Zimmermann, Emily G. Barr Fritcher, Christopher D. Zysk, Rory A. Jackson, Asha A. Nair, Robert B. Jenkins, Kevin C. Halling, Benjamin R. Kipp, Cristiane M. Ida

**Affiliations:** 1 Department of Laboratory Medicine and Pathology, Mayo Clinic, 200 First St SW, Rochester, MN 55905 USA; 2 Department of Health Sciences Research, Mayo Clinic, Rochester, MN USA; 3 Department of Pathology, Dell Children’s Medical Center, Austin, TX USA; 4 Neuro-Oncology Division, Dell Children’s Medical Center, Austin, TX USA; 5 Bioinformatics Research and Development Laboratory, Genomics Sciences and Precision Medicine Center, Medical College of Wisconsin, Milwaukee, WI USA

**Keywords:** BRAF, DIG, MAPK, Targeted therapy

Desmoplastic infantile ganglioglioma (DIG) and desmoplastic infantile astrocytoma (DIA) are rare, low-grade neuroepithelial neoplasms [1]. *BRAF* alterations, primarily the V600E mutation and rarely V600D and *FXR1*-*BRAF* fusion [3–5, 8, 10, 12], have been described for DIG/DIA. Although gross total resection is typically curative, tumor location may prevent complete tumor excision. Additionally, tumor recurrence, progression, and rarely leptomeningeal dissemination have been reported [2, 9], underscoring the need for adjuvant treatment.

With comprehensive molecular analysis, we identified a novel *BRAF* alteration in a DIG in a 3-month-old female patient who had seizures, apnea, and a right postcontrast enhancing temporal solid multicystic mass (Fig. 1a). Three months after near-total tumor resection, progressive brainstem leptomeningeal spread (Fig. 1b) prompted a second operation (near-total completion). The tumors from both resections were histologically similar: A prominent desmoplastic stroma had astrocytic, neoplastic neuronal, and poorly differentiated neuroepithelial tumor cell components (Fig. 2). Mitotic activity (up to 6/10 high-power fields) was limited to the poorly differentiated neuroepithelial component. Neither necrosis nor microvascular proliferation was observed.

**Fig. 1:**
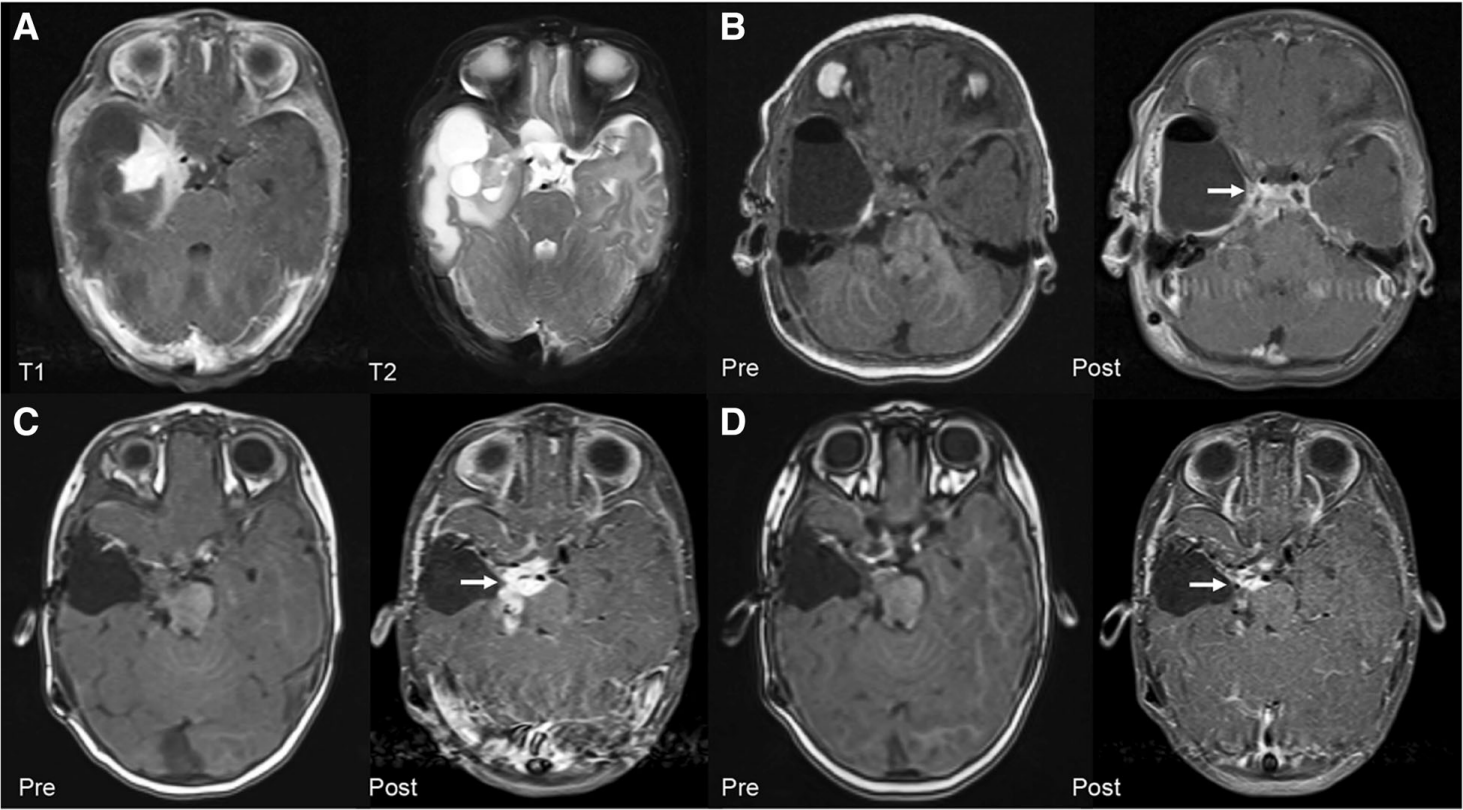
Radiologic findings. **a** (T1-Weighted Postcontrast and T2-Weighted Axial Magnetic Resonance Imaging), Large right inferomedial temporal solid-multicystic mass with a postcontrast enhancing component. **b** (T1-Weighted Pre and Postcontrast Axial Magnetic Resonance Imaging), Three-month postoperative leptomeningeal spread involving the upper brainstem. **c** (T1-Weighted Pre and Postcontrast Axial Magnetic Resonance Imaging), Eight-month postoperative progression of leptomeningeal involvement despite standard chemotherapy. **d** (T1-Weighted Pre and Postcontrast Axial Magnetic Resonance Imaging), Fourteen-month postoperative decrease in residual tumor after 6 months of BRAF-MEK inhibitor therapy

**Fig. 2:**
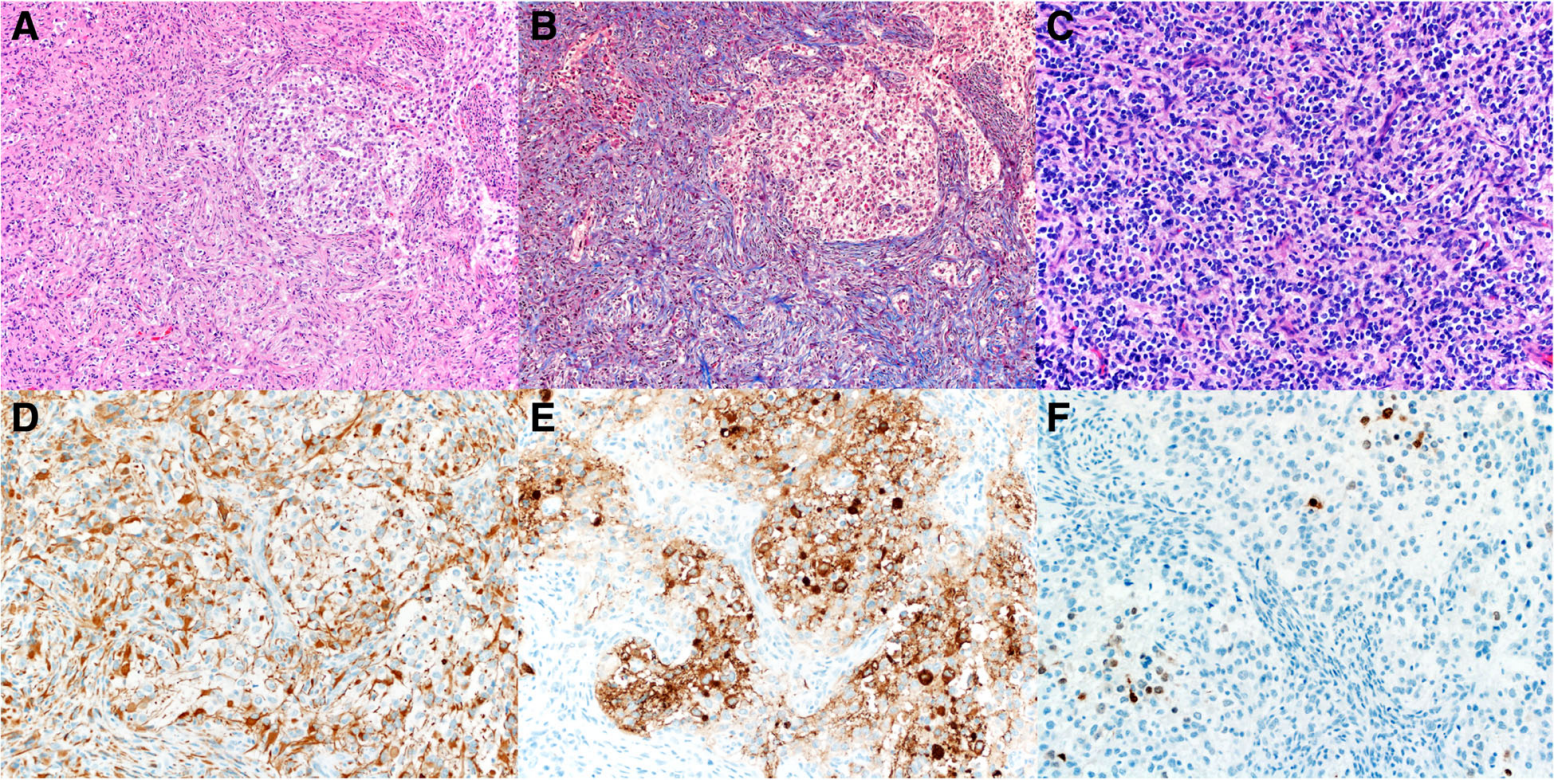
Histologic findings. **a**, Astrocytic and neoplastic neuronal tumor cell components (hematoxylin-eosin, × 100). **b** Prominent desmoplastic stroma (Masson’s trichrome, 100x). **c** Focal poorly differentiated neuroepithelial (small cell) component (hematoxylin-eosin, × 200). **d** Glial fibrillary acidic protein immunostain highlighting the astrocytic tumor cell component (× 200). **e** Synaptophysin and **f** Neu-N immunostain highlighting the neoplastic neuronal tumor cell component (× 200)

Comprehensive molecular tumor profiling was performed with a 150-gene DNA and an 81-gene RNA neurooncology next-generation sequencing panel (Additional file 1: Methods). A *BRAF* indel involving codons 600–604 (c.1799_1810delinsACCAAACTGATG; p.V600_W604delinsDQTDG) at low variant allelic frequency (approximately 15%) was the only clinically relevant alteration identified (Additional file 2: Figure S1). This alteration was confirmed with Sanger sequencing (Additional file 3: Figure S2), and mRNA expression was demonstrated with RNA sequencing (Additional file 4: Figure S3). In silico protein modeling (Additional file 1: Methods) with wild-type, pS602, and V600E comparators showed that the novel *BRAF* indel had the greatest positional change compared with wild-type, which was consistent with stabilization of the kinase-active conformation (Fig. 3 and Additional file 5: Figure S4).

**Fig. 3:**
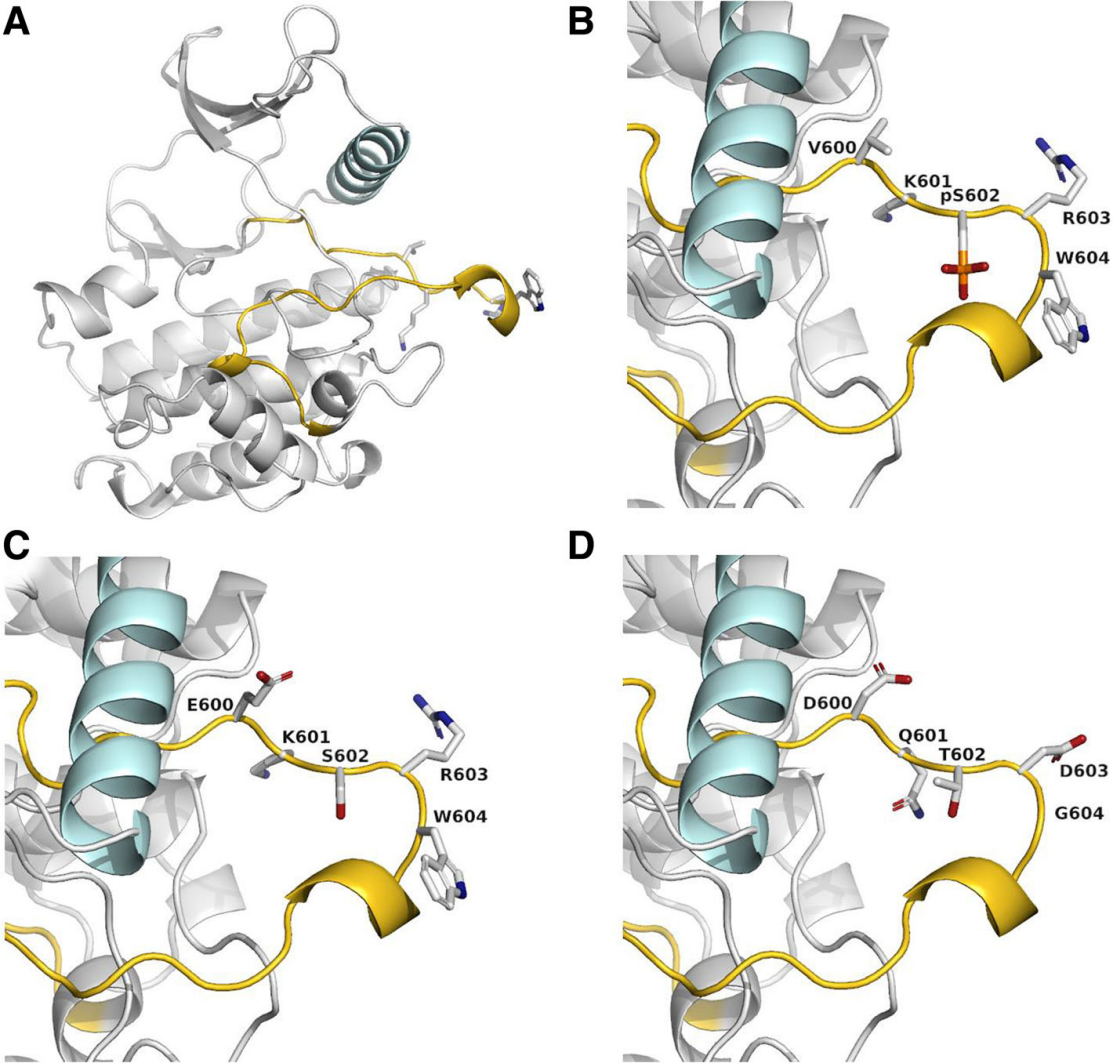
In Silico Protein Modeling. **a** Wild-type BRAF kinase domain. Cyan indicates αC helix; gold, activation loop. Residues affected by novel indel are colored according to type of atom. **b** Normal activation mechanism of wild-type BRAF protein. **c** *BRAF* V600E constitutively active variant. **d** Novel BRAF indel with changes to numerous amino acids in the kinase domain activation loop, consistent with a kinase-active conformation

Postoperatively, vincristine and carboplatin chemotherapy was initiated upon disease progression. Despite treatment, the leptomeningeal lesions continued to progress (Fig. 1c), and treatment was switched to BRAF-MEK inhibitors (dabrafenib and trametinib) at 8 months postoperatively. The patient is alive with marked decrease in residual tumor and leptomeningeal disease 14 months after the initial surgery (Fig. 1d).

Comprehensive tumor molecular profiling led to the discovery of a novel *BRAF* alteration, increasing the number of *BRAF* alterations identified in DIG/DIA. The oncogenic role of this novel *BRAF* alteration is supported by the protein modeling and by the observed clinical response to BRAF-MEK inhibitors. This finding suggests that, like other low-grade neuroepithelial tumors [6, 7, 11], mitogen-activated protein kinase (MAPK) pathway activation may have a potential oncogenic-driver role in a subset of patients with DIG/DIA. After complete DIG/DIA resection, patients typically have a favorable outcome regardless of the histologic features. Dissemination, as in our patient, is exceedingly rare, and no histologic or molecular parameters are currently predictive of a less favorable outcome [1]. Although additional studies are needed, the responsiveness to BRAF-MEK inhibitors in a DIG with a novel, likely oncogenic *BRAF* alteration suggests that routine molecular testing for this rare pediatric tumor may be part of a personalized medicine approach, particularly when gross total resection is not achieved and adjuvant therapy is considered.

## Additional files

Additional file 1: Supplementary methods. (DOCX 37 kb)

Additional file 2: DNA NGS results. (TIF 699 kb)

Additional file 3: Sanger sequencing results. (TIF 522 kb)

Additional file 4: RNA sequencing results. (TIF 443 kb)

Additional file 5: In silico protein modelling. (TIF 690 kb)

## Abbreviations

DIA: Desmoplastic infantile astrocytoma
DIG: Desmoplastic infantile ganglioglioma
MAPK: Mitogen-activated protein kinase
