# Supplementary material for: Novel BRAF alteration in desmoplastic infantile ganglioglioma with response to targeted therapy
Source: Acta Neuropathol Commun. 2018 Nov 5;6:118. doi: 10.1186/s40478-018-0622-1 (PMC6219207; doi:10.1186/s40478-018-0622-1)
Supplement: Supplementary file 1 — Supplementary methods. (DOCX 37 kb) [file 40478_2018_622_MOESM1_ESM.docx]

**Supplementary Methods**

Formalin-fixed paraffin embedded (FFPE) tumor tissue was macrodissected for tumor enrichment from 5-micron unstained sections. The macrodissected tissue included approximately 90% tumor and 10% non-neoplastic brain based on evaluation of the matched hematoxylin-eosin-stained section. The neuroepithelial (neoplastic glial, neuronal and poorly differentiated) component of the tumor was estimated to be approximately 30 to 40%, based on immunohistochemical studies using GFAP (polyclonal, 1:8000; Dako, Glostrup, Denmark), synaptophysin (clone 27G12, 1:50; Leica, Newcastle Upon Tyne, United Kingdom) and Neu-N (clone A60, 1:100; Zeta Corp., Arcadia, CA) performed following clinically validated protocols. DNA was extracted using the QIAamp DSP DNA FFPE Tissue Kit (Qiagen Inc., Germantown, MD) with few modifications [2]. RNA was extracted using the Qiagen miRNeasy Micro Kit. RNA was converted to cDNA using the QIAseq Targeted RNAscan panel and the SureSelect XT RNA Direct Kit (Agilent Inc., Santa Clara, CA) for the RNA panel and the RNA sequencing, respectively.The DNA panel NGS library was prepared using a QIAseq Targeted DNA custom amplicon-based panel designed to interrogate 150 central nervous system (CNS) tumor-associated genes (Supplementary Table 1). The RNA panel NGS library was prepared using a QIAseq Targeted RNAscan custom amplicon-based panel (Supplementary Table 2) designed to detect 104 known gene-gene fusions, 29 abnormal transcript variants, and novel fusion transcripts containing one gene partner from the CNS tumor-associated 81-gene primer pool. The RNA sequencing NGS library was prepared using an Agilent SureSelect XT RNA Direct custom panel that evaluates 2,360 cancer-related genes (gene list available upon request). Paired-end 2x151 base-pair sequencing were performed on an Illumina HiSeq 2500 instrument (Illumina, Inc., San Diego, CA) for the DNA panel and RNA sequencing. Paired-end 231/71 sequencing was performed on an Illumina MiSeq instrument for the RNA panel. DNA and RNA panel data were processed through custom bioinformatics pipelines developed to detect single nucleotide variants and small insertions/deletions (< 50 base pairs) with at least 15% variant allelic frequency and gene fusion events with at least 10 fusion transcripts including 5 unique molecules, respectively. DNA and RNA sequence alterations were visualized using Alamut visual, version 2.7 rev. 2 (Interactive Biosoftware, Rouen, France) and integrative genome browser (IGV) [5], respectively. DNA alterations showing a minimum of 100x depth of coverage were reviewed and classified as benign polymorphism, variant of unknown significance or pathogenic mutation based on publicly available genetic databases and literature.

Targeted direct Sanger sequencing was performed using primers with custom 5-prime universal primer sequencing (UPS) tails designed to flank the region including the novel *BRAF* alteration detected by NGS (forward: 5’UPS-GACCTTCAATGACTTTCTAGTAACTCAGC3’; reverse: 5’UPS-ACCTAAACTCTTCATAATGCTTGCTCT3’). Template PCR was performed using the KAPA2G PCR system (KAPA Biosystems, Woburn, MA). PCR amplicons were sequenced bi-directionally, using the BigDye Terminator v1.1 cycle sequencing kit (Applied Biosystems, Thermo Fisher Scientific, Foster City, CA). Diluted purified sequencing products were detected on the ABI 3730xl DNA Analyzer capillary electrophoresis instrument. Sequencing traces were analyzed using the Mutation Surveyor software (Soft Genetics, State College, PA).

In-silico protein modeling was developed to confirm enzyme conformation change and to predict pathogenicity. Three comparators -WT, pS602, and V600E- were used. We defined the activation loop (a-loop) as residues between the DFG and MAP motifs; residues 594 through 622. The αC helix is the only helix in the N-terminal lobe of the kinase domain; residues 492 through 507. The HRD motif is within the catalytic loop and nearby in space to the activation loop. We assessed changes in protein dynamics using implicit solvent molecular dynamics (MD) simulations using NAMD [3]. Each sequence was modeled and independent triplicate simulations generated. Each was energy minimized for 10,000 steps, followed by gradual heating to 300K over 0.6ns, equilibration for 12.4ns, and a further 10ns of simulation generated for analysis. Analysis was carried out using custom scripts leveraging VMD and the bio3D R package [1]. Protein structures were visualized using PyMOL [4] and VMD v1.9.3. We calculated Root Mean-Squared Fluctuation (RMSF) and Principal Component (PC) analysis using C^α^ atoms in Cartesian space.

**References**

1. Grant BJ, Rodrigues AP, ElSawy KM, McCammon JA, Caves LS (2006) Bio3d: an R package for the comparative analysis of protein structures. Bioinformatics 22:2695-2696. doi:10.1093/bioinformatics/btl461

2. Ida CM, Vrana JA, Rodriguez FJ, Jentoft ME, Caron AA, Jenkins SM, Giannini C (2013) Immunohistochemistry is highly sensitive and specific for detection of BRAF V600E mutation in pleomorphic xanthoastrocytoma. Acta Neuropathol Commun 1:20. doi:10.1186/2051-5960-1-20

3. Phillips JC, Braun R, Wang W, Gumbart J, Tajkhorshid E, Villa E, Chipot C, Skeel RD, Kale L, Schulten K (2005) Scalable molecular dynamics with NAMD. J Comput Chem 26:1781-1802. doi:10.1002/jcc.20289

4. PyMOL (2018) The PyMOL Molecular Graphics System. Version 1.7.6. Schrödinger, LLC. Available from: https://pymol.org/2/. Accessed Aug 7 2018

5. Thorvaldsdottir H, Robinson JT, Mesirov JP (2013) Integrative Genomics Viewer (IGV): high-performance genomics data visualization and exploration. Brief Bioinform 14:178-192. doi:10.1093/bib/bbs017

| **Supplementary Table 1.** DNA gene panel list (genes in bold also evaluated by the RNA panel) | | | | | | | |  |  |
| --- | --- | --- | --- | --- | --- | --- | --- | --- | --- |
| *ACAN* | *CACNG6* (promoter only) | *DNMT3A* | *GPS2* | *KDM6A* | *MSH2* | *OTX2* | *PTCH1* | *SETD2* | *TERT* (with promoter) |
| *ACVR1* | *CBL* | *EEF1A1* | *H3F3A* | *KDM6B* | *MSH3* | *PARP1* | *PTCH2* | *SHH* | *TET1* |
| *AKT1* | *CDK6* | ***EGFR*** | *HDAC2* | *KEL* | *MSH6* | ***PDGFRA*** | *PTEN* | *SHOC2* | *TET2* |
| *AKT2* | *CDKN2A* | *EZH2* | *HDAC9* | *KLF4* | *MUC17* | *PIK3C2B* | *PTPN11* | *SMARCA4* | *TP53* |
| *AKT3* | *CDKN2B* | *F5* | *HIST1H3B* | *KMT2B* | ***MYB*** | *PIK3C2G* | *PTPRD* | *SMARCB1* | *TPTE2* |
| *AOX1* | *CDKN2C* | ***FGFR1*** | *HIST1H3C* | *KMT2C* | ***MYBL1*** | *PIK3CA* | ***QKI*** | *SMARCE1* | *TRAF7* |
| *APC* | *CHEK2* | *FGFR2* | *HMCN1* | *KMT2D* | ***MYC*** | *PIK3R1* | ***RAF1*** | *SMO* | *TRIM28* (promoter only) |
| *ARID1A* | *CIC* | ***FGFR3*** | *IDH1* | *KRAS* | *MYCN* | *PIK3R2* | *RB1* | *SNCAIP* | *TRPA1* |
| *ARID2* | *COL6A3* | *FUBP1* | *IDH2* | *LAMA4* | *NF1* | *PLCG1* | ***RELA*** | *SOS1* | *TSC1* |
| *ATM* | *CREBZF* | *GABRA6* | *IL4R* | *LDB1* | *NF2* | *PMS1* | *RPL5* | *STAG2* | *TSC2* |
| *ATRX* | *CSNK2B* | *GLI2* | *JAK2* | *LRP1B* | *NIPBL* | *POLE* | *SDHA* | *STAT3* | *WRN* |
| *BAP1* | *CTDNEP1* | *GLI3* | *KDM4D* | *LZTR1* | *NOTCH1* | *POLR2A* | *SDHB* | *SUFU* | *WT1* |
| *BCOR* | *CTNNB1* | *GNA11* | *KDM5A* | *MAP2K1* | *NOTCH2* | *POT1* | *SDHC* | *TCF12* | ***YAP1*** |
| *BCORL1* | *DAXX* | *GNAQ* | *KDM5B* | *MDM2* | *NRAS* | *PPM1D* | *SDHD* | *TEP1* | *ZBTB20* |
| ***BRAF*** | *DDX3X* | *GNAS* | *KDM5C* | *MLH1* | *NUP210L* | *PRKAR1A* | *SEMG1* | *TERF1* | *ZMYM3* |

**Supplementary Table 2.** RNA gene panel list (genes in bold also evaluated by the DNA panel)

| *AFAP1* | *CLCN6* | *FAM118B* | *GLI1* | *MN1* | *NFASC* | *PTPRZ1* | *SRGAP3* | ***YAP1*** |
| --- | --- | --- | --- | --- | --- | --- | --- | --- |
| *AGBL4* | *CLIP2* | *FAM131B* | *GNAI1* | *MST1R* | *NRF1* | *PVT1* | *ST6GAL1* |  |
| *ATG7* | *CXXC5* | ***FGFR1*** | *JPX* | ***MYB*** | *NTRK1* | ***QKI*** | *STAT6* |  |
| *BCAN* | *DDX31* | ***FGFR3*** | *KIAA1549* | ***MYBL1*** | *NTRK2* | ***RAF1*** | *TACC1* |  |
| *BEND2* | *DIP2C* | *FLI1* | *LOC550643* | ***MYC*** | *NTRK3* | *RECK* | *TACC3* |  |
| *BIRC5* | ***EGFR*** | *FOXR2* | *MACF1* | *NAB2* | *PCDHGA1* | ***RELA*** | *TFG* |  |
| ***BRAF*** | *ELAVL3* | *FXR1* | *MAMLD1* | *NACC2* | *PCSK5* | *RNF130* | *TPM3* |  |
| *BTBD1* | *ESR1* | *FYCO1* | *MET* | *NAV1* | ***PDGFRA*** | *SEPT14* | *UBE2J2* |  |
| *C11orf95* | *ETV6* | *GFI1* | *MKRN1* | *NDRG1* | *PKD1* | *SLC44A1* | *VCL* |  |
| *C8orf34* | *EWSR1* | *GFI1B* | *MMP16* | *NELFE* | *PRKCA* | *SLIT1* | *WHSC1* |  |

**Supplementary Figure Legends**

**Supplementary Figure** **1.** Sequencing reads showing the novel *BRAF* indel (NM_004333.4:c.1799_1810delinsACCAAACTGATG; p.Val600_Trp604delinsAspGlnThrAspGly). Per HGVS nomenclature, this alteration would be described as: (NM_004333.4:c.[1799_1801delinsACC;1804T>A;1807del;1812dup]) detected by 150-gene DNA NGS panel.

**Supplementary Figure 2.** Electropherogram traces confirming the novel *BRAF* indel (NM_004333.4:c.1799_1810delinsACCAAACTGATG; p.Val600_Trp604delinsAspGlnThrAspGly) by Sanger sequencing.

**Supplementary Figure 3.** Sequencing reads demonstrating the novel *BRAF* indel (NM_004333.4:c.1799_1810delinsACCAAACTGATG; p.Val600_Trp604delinsAspGlnThrAspGly) at the mRNA level by RNA sequencing.

**Supplementary Figure 4.** Distance between the BRAF HRD motif and residue 600 in the activation loop. The distance from the Cα atom of R575 in the HRD motif to the Cα atom of V600 or E600 was monitored as a summary metric for the activation loop moving to an activated position. Compared to wild-type (WT), all three contexts -V600E, pS602 (Phos) and novel indel (DQTDG) showed an increased distance.
