# Supplementary figures and images for: Novel BRAF alteration in desmoplastic infantile ganglioglioma with response to targeted therapy
Source: Acta Neuropathol Commun. 2018 Nov 5;6:118. doi: 10.1186/s40478-018-0622-1 (PMC6219207; doi:10.1186/s40478-018-0622-1)

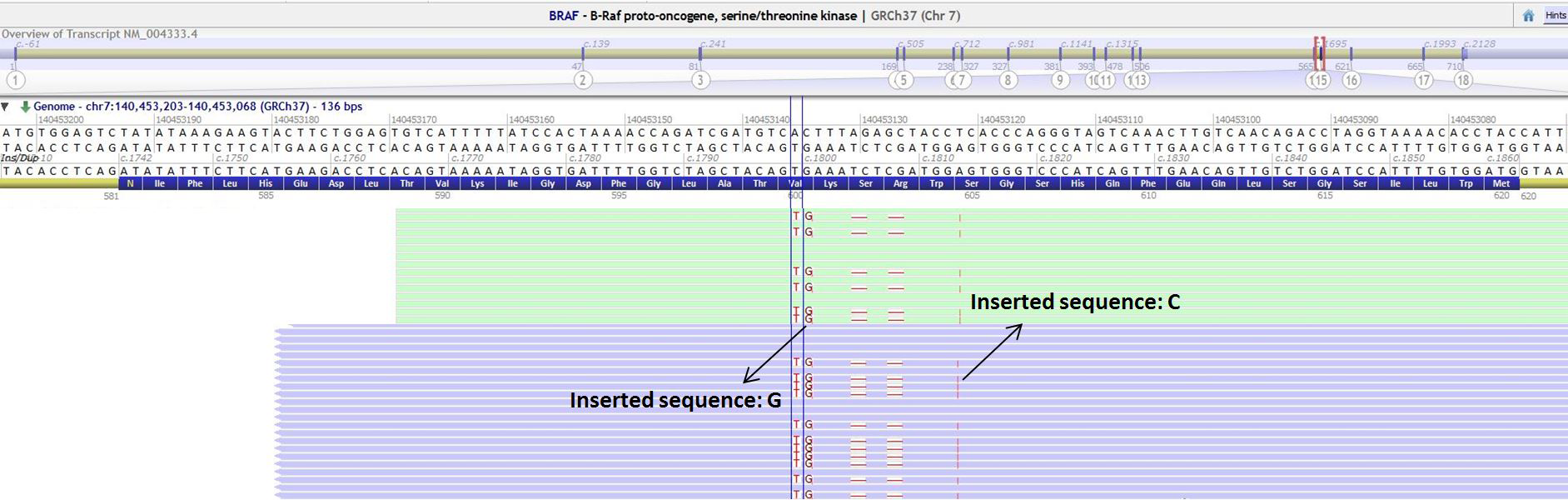

Supplement: Supplementary file 2 — DNA NGS results. (TIF 699 kb) [file 40478_2018_622_MOESM2_ESM.tif]

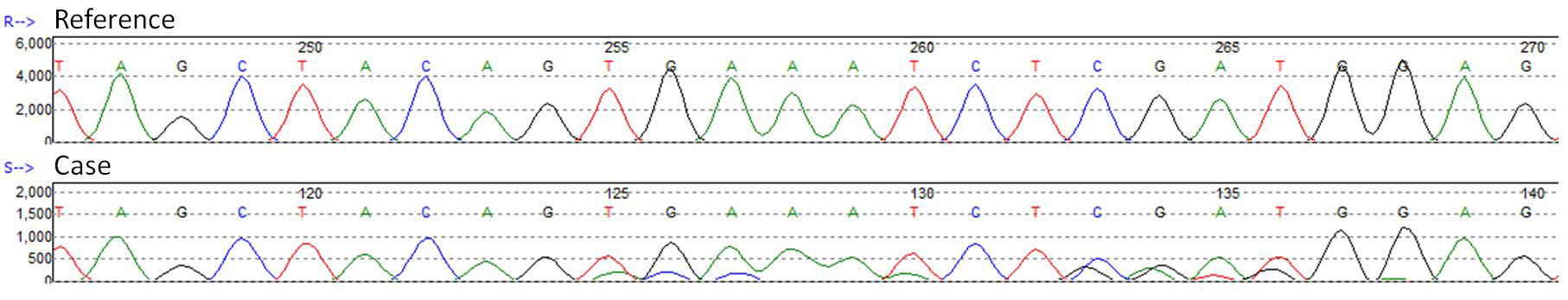

Supplement: Supplementary file 3 — Sanger sequencing results. (TIF 522 kb) [file 40478_2018_622_MOESM3_ESM.tif]

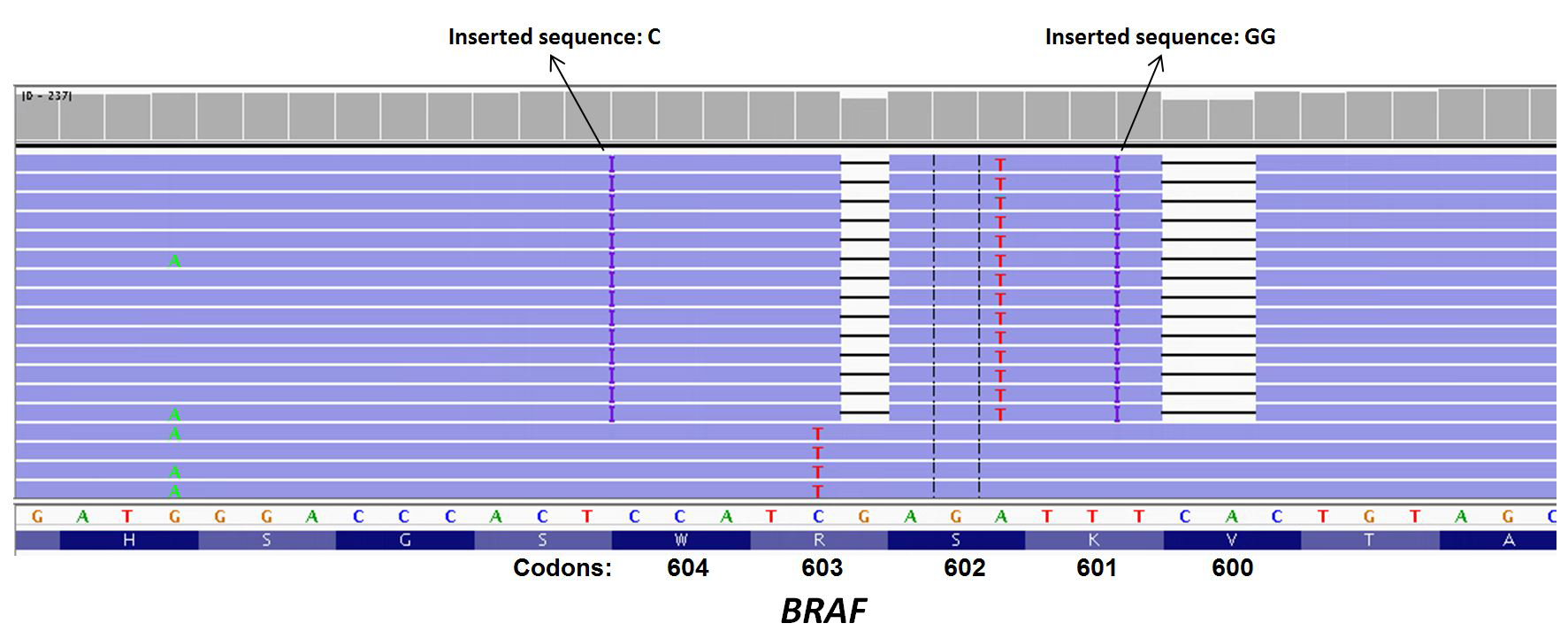

Supplement: Supplementary file 4 — RNA sequencing results. (TIF 443 kb) [file 40478_2018_622_MOESM4_ESM.tif]

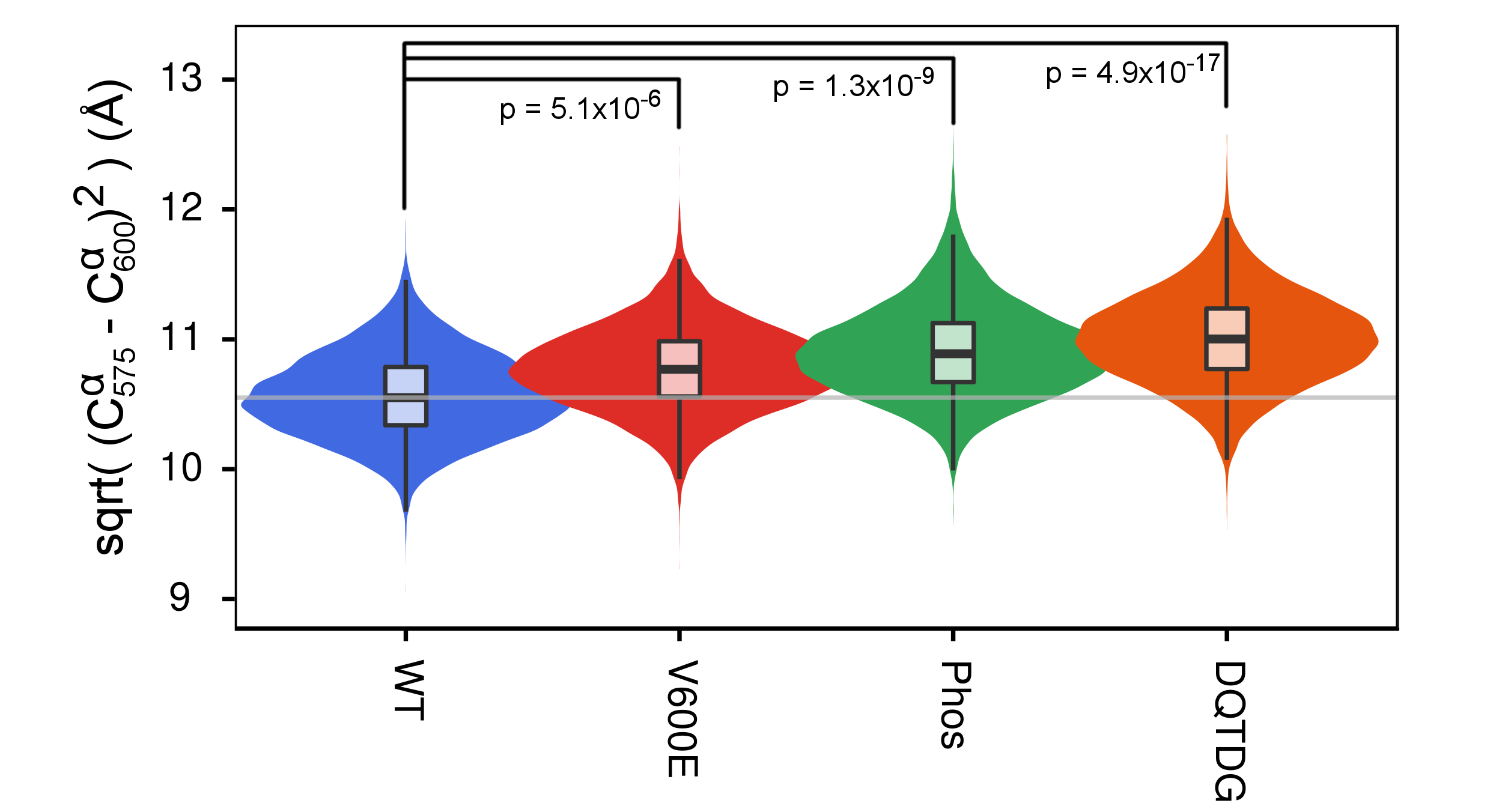

Supplement: Supplementary file 5 — In silico protein modelling. (TIF 690 kb) [file 40478_2018_622_MOESM5_ESM.tif]
